# Nanoscale Melting of 3D Confined Azopolymers through Tunable Thermoplasmonics

**DOI:** 10.1021/acs.jpclett.2c01103

**Published:** 2022-06-09

**Authors:** Sergey S. Kharintsev, Sergei G. Kazarian

**Affiliations:** † Department of Optics and Nanophotonics, Institute of Physics, Kazan Federal University, Kremlevskaya, 16, Kazan 420008, Russia; ‡ Department of Chemical Engineering, Imperial College London, South Kensington Campus, SW7 2AZ London, United Kingdom

## Abstract

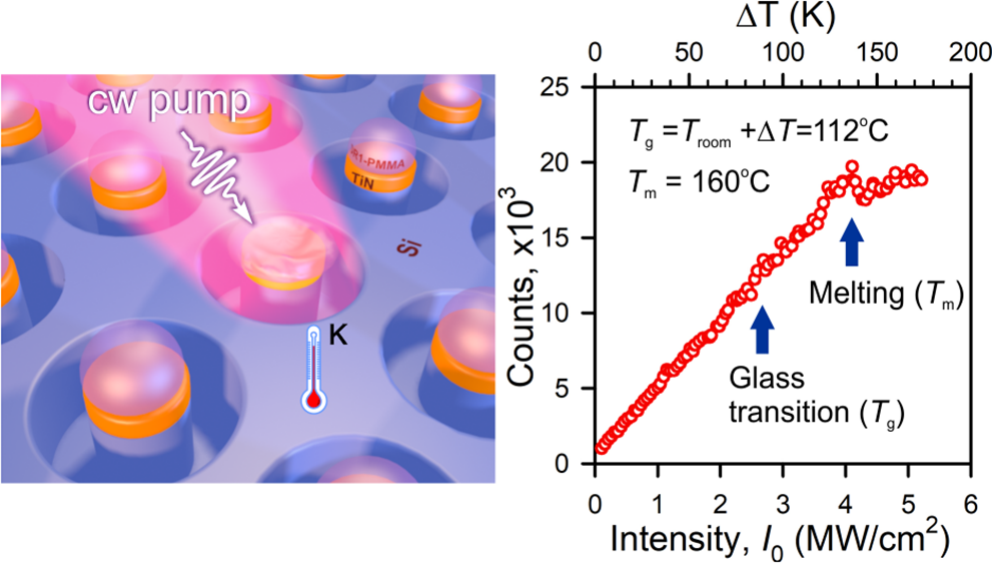

Phase transitions that are thermally induced by using light at the nanoscale play a vital role in material science. Enhanced optical heating sustained by resonant nanostructures can turn out to be insignificant when a higher thermal conductivity of a heatsink, regardless of the pumping intensity. In this Letter, we demonstrate an approach to control an operating temperature range due to excess heating of a structured heatsink. A design rationale has been performed by using a 2D array of TiN:Si voxels, consisting of stacked TiN and Si pillars. All the TiN nanoheaters responsible for enhanced light absorption at plasmon resonance are of equal size, and the height of the Si pillars varies to control heat localization. A height-dependent temperature rise of the Si pillars is found from Raman thermometry. Herein, for the first time, we have determined the melting temperature of azobenzene-functionalized polymers at the nanoscale using the tunable plasmonic metasurface.

Ongoing progress in nanofabrication tools inevitably challenges characterization methods of spatially limited materials because their physical and chemical properties can be noticeably different from those of the bulk. Size effects of one-of-a-kind parts play a crucial role in the miniaturization of diverse functional micro- and nanodevices ranging from optical metamaterials to biomedical laboratories on a chip.^1−3^ For example, a two-photon polymerization direct laser writing technique enables patterns of polymeric structures with sizes of <50 nm to be achieved.^4,5^ These constituents may behave differently even under conventional conditions; therefore, the development of unique analytical sensors for probing the physical–chemical properties of tiny topologies is of keen interest in the nanofabrication industry.

One of the impressive effects in condensed-matter physics is the variation of the glass transition temperature *T*_g_ when lowering the size of low-molecular-weight and polymeric glass-formers.^6^ Depending on the strength of sample–substrate interactions, these characteristics can be either increased or decreased.^7,8^ These effects have been well studied for two-dimensional polymeric structures—thin films in which only their thickness varies.^9−11^ However, lower dimensional topologies, such as polymeric rods and dots, have not been investigated to the best of our knowledge. This is probably because of the lack of proper analytical instruments to probe the physical and chemical properties of 3D confined polymers. The use of the latter as building blocks in a lab-on-a-chip device requires their resistance to higher working temperatures. This circumstance has gained interest in the synthesis of cross-linked polymeric networks with higher *T*_g_’s to maintain a nanoscopically confined former unchanged.

To date, there have been two promising sensors developed based on a plasmonic metasurface concept to recognize the *T*_g_.^12,13^ One of them represents a 3D plasmonic sensor consisting of a quasi-random array of designed cones. Each cone represents a composite structure of two silver disks of different diameters spaced by a SiO_2_ truncated cone.^12^ This design maintains two spectrally well-separated localized plasmon resonances (LPRs) at the lower and upper disks. Upon heating a specimen of interest by using Ar blowing, its local refractive index *n* decreases due to thermal expansion; as a result, both the LPRs are shifted. With the advent of the glass transition, a free volume of the polymer increases sharply, and a hallmark kink in *n* is observed. This footprint allows quantification of the *T*_g_. The advantage of such 3D plasmonic sensor is what to probe the glass transition temperature both laterally and axially.

Another approach is underpinned on a tunable optical heating of an ordered two-dimensional (2D) array of stacked TiN and Si pillars; it is further referred to as a 3D TiN:Si voxel due to reinforced optical absorption of a TiN pad at plasmon resonance and heat localization within a Si pillar.^13,14^ Each 3D voxel is heated independently when it is illuminated by a focused CW laser light. Importantly, a net temperature rise is dependent on not only absorbed energy but also excess heating of a designed heatsink. This means that the optical heating can be directly tuned by the height of Si pillars rather than the pump power.^13^ In the context of a 2D plasmonic sensor, the height controls an operational temperature range, whereas the pumping intensity does on-demand temperature. At the *T*_g_ event, the defrosting of vibrational and rotational modes results in the enhancement of Raman scattering that simultaneously plays a role of a glass-to-rubber transition indicator and a remote thermometer. In addition, the TiN pad serves as an optical nanoantenna enabling an enhanced Raman response many times over.^15^

In this Letter, we demonstrate the potential of a 2D array of TiN:Si voxels for optical probing of the local melting of poly[(methyl methacrylate)-*co*-(Disperse Red 1 methacrylate)] nanostructures. The operational temperature range is set by varying the height of the Si pillars, whereas the temperature increment itself is managed by the pumping intensity within this range. Upon reaching the melting point, *T*_m_, the amount of the melted polymer diminishes due to its reduced viscosity and a limited lateral size of the TiN:Si voxel. The depletion of a Raman signal of the polymer with increased pump power is a strong signature to recognize the melting temperature. The merit of this method aims at the tunable temperature range at a given pumping intensity and a small relaxation time (of <1 μs^16^) to reach a thermal equilibrium of the nanoheater. The proposed approach allows, for the first time to the best of our knowledge, the first-order phase transitions of 3D confined polymers at the nanoscale to be probed.

Boltzmann heating solids, due to their light absorption at the subwavelength scale, are still one of the up-and-coming research interests in thermo-nanophotonics.^16−20^ An underlying challenge is linked to weak light–matter interactions by understanding that the size of a radiating dipole (*l*) is much less than the light wavelength (λ) by roughly 3 orders of magnitude. Light energy absorbed by a nanostructure under CW illumination with the pumping intensity *I*_0_ is entirely transferred to heat, and thus the heat source power is
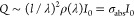
1where ρ(λ) is a local density of states (LDOS) and σ_abs_ is an absorption cross section. Equation 1 is true provided that light scattering and reflection are negligible. A sole possible route to boost optical heating of an absorbing nano- and microstructure is the increase of the LDOS through the excitation of resonant modes. For example, the LDOS is considerably enhanced due to localized plasmon resonances (LPRs) supported with a metallic nanoparticle.^16,18,20^ Under a dipole plasmon resonance subject to the Fröhlich condition Re[ε(λ_res_)] = −2ε_s_ (λ_res_ is the resonant wavelength), a temperature increment of the metallic nanostructure exposed to CW illumination reads^16,18^
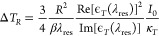
2Here ε_*T*_(λ) is the temperature- and wavelength-dependent permittivity of the metallic nanostructure, κ_*T*_ is the temperature-dependent thermal conductivity of the surrounding, β is a dimensionless thermal capacitance coefficient, depending on the nanostructure geometry, and *R* is the radius of a sphere with the same volume as the nanostructure.^16^ At a resonance, the heating of a nanostructure is controlled by the pump power *I*_0_ and the thermal conductivity κ_*T*_ only. This means that the optical heating of a nanostructure surrounded by a high thermal conductivity thermostat (κ_*T*_ → ∞) is negligible regardless of a pumping intensity. At a fixed pump power, the tunable optical heating can be performed through heat localization within a spatially limited designed heatsink, as schematically shown in Figure 1a. When a heat-generated nanostructure is placed on a rod-shaped heatsink of height *h*, the temperature increase of the former is determined by the following formula (see details in the Supporting Information)
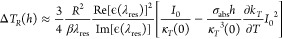
3where κ_*T*_(0) is the temperature-dependent thermal conductivity of the heatsink at *h* = 0. The second term in eq 3 has a negative sign because ∂*k*_*T*_/∂*T* < 0 for the most materials at room temperature.

**Figure 1:**
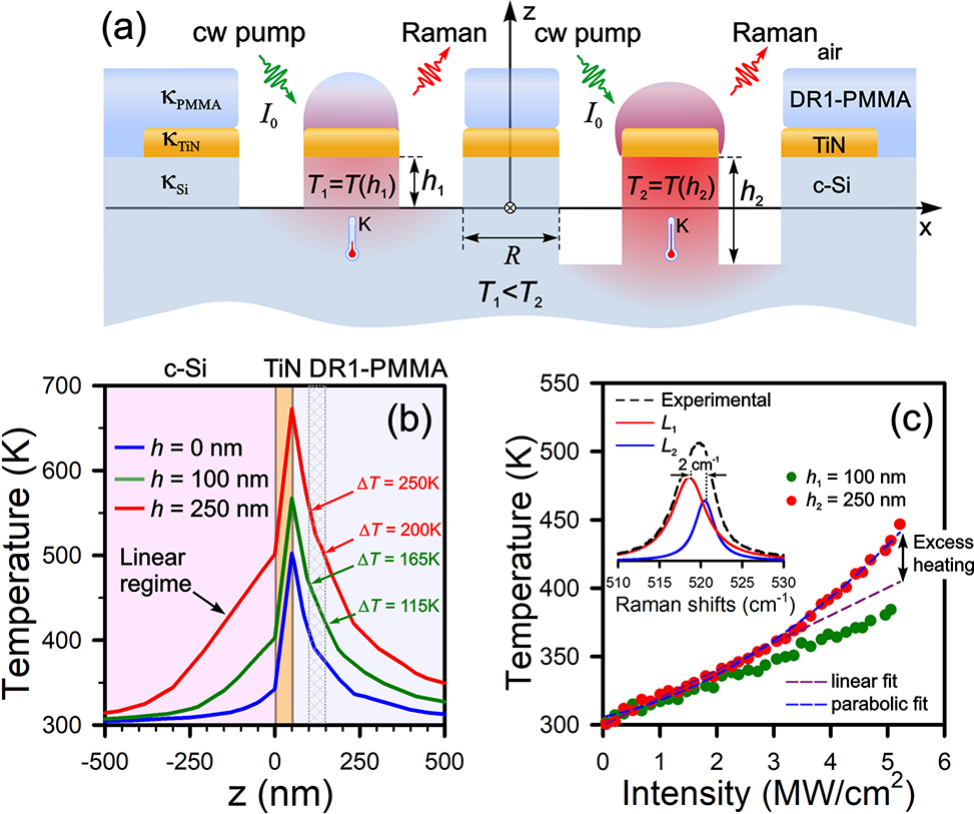
(a) Schematic of a 2D array of TiN:Si voxels capped with polymer pads (side view). (b) FDTD calculated temperature profile across the *z*-axis. (c) A measured temperature vs pumping intensity for the different heights; the inset shows a Raman spectrum of the Si (dashed curve) decomposed into two elementary Lorentzians (a red/blue curve corresponds to a hot/cold contribution).

Figure 1a shows a schematic (side view) of a 2D array of stacked a titanium nitride (TiN) pad (plasmonic nanoheater) and a silicon (Si) pillar (1D heatsink); it is referred to as a 3D TiN:Si voxel. Under focused CW laser light, the TiN pad is heated to high temperatures due to the plasmon-enhanced absorption. The one-dimensional Si heatsink enables the outflow of heat toward the thermostat to be slowed. By variation of the height of the Si pillar, it can settle on-demand at an operational temperature range, within the limits of which the temperature can be smoothly controlled with the pumping intensity *I*_0_. This design rationale can be used to optically probe phase transitions at the nanoscale. To understand this point, let us numerically calculate the temperature *z*-profile across the polymer-capped TiN:Si voxel using FDTD/FEM simulations. Figure 1b features three *z*-profiles for different heights: 0, 100, and 250 nm. With increasing height, we observe the linear regime of a thermal gradient inside the Si pillar; this witnesses a slowed outflow of heat and further the increase of temperature on the bottom and top surfaces of the TiN pad. The polymer size (the film thickness) is of importance because the temperature drops according to a fractional power law.^13^ Temperature gradients inside the shaded region of 50 nm are equal to 1 K/nm for the heights of 100 nm (green) and 250 nm (red). This indicates that not only the chemical nature of a polymer but also its size plays an important role for choosing the height of Si pillars. It is feasible to fabricate a thermal metasurface with different heights of Si pillars to find the ones that best fit the sample. Based on simulations, to melt a polymer with the thickness of 50 nm at *T*_m_ ≈ 160 °C, the height of a Si pillar should be not less 100 nm (Figure 1b).

In our experiment the temperature rise of the polymer-capped TiN:Si voxel is measured by using confocal Raman thermometry.^13,21^ To exclude a unwanted vibrational (non-Boltzmann) pumping,^22^ we utilize a Raman shift-based probe that provides a unprecedented high spectral resolution of 0.1 cm^–1^ due to an Echelle grating. This means that a temperature accuracy is estimated to be around 5 K. Herein, we used the first-order Raman peak of Si at 521 cm^–1^ as a reliable temperature-dependent Raman reporter (a calibration plot is given in Figure S1). The temperature increment of a polymeric nanostructure itself is calculated from FDTD/FEM simulations using *a priori* information about a measured temperature of a Si pillar.

Figure 1c shows a plot of the temperature increment vs pumping intensity for two heights of the Si pillars: 100 nm (green circles) and 250 nm (red circles). The former exhibits a linear trend in full correspondence to eq 2, whereas the latter has a parabolic behavior (eq 3). The nonlinear deviation of the temperature increment can be understood through the temperature-dependent thermal conductivity of Si that is decreased with increasing temperature. This leads to an excess heating, as displayed in Figure 1c. It is important to realize that monitoring the Raman shift of a hot spot from Si is not a trivial task because it is overlapped with that of a cold spot. This fundamental challenge is caused by the nonuniform broadening that forms the continuum of Raman peaks originating from gradually heated silicon extents in the vicinity of the hot spot. The uncertainty, which plays an essential role at a weak pumping, can be eliminated by decomposing the Raman band into two elementary Lorentizians *L*_1_ and *L*_2_ attributed to “hot” and “cold” Si, respectively, as shown in the inset of Figure 1c. The deconvolution is performed by using a regularized least-squares method.^23^ This plot confirms that the error can achieve 2 cm^–1^ or even worse, and thus the temperature accuracy can raise up to 100 K or above.

According to eq 3, we fit the experimental data (red circles in Figure 1c) using a parabolic function (a blue dashed curve) with χ^2^ = 0.997. Though, prior to 3 MW/cm^2^, a linear trend (a dashed lilac curve) with χ^2^ = 0.995 is obvious. The second term in eq 3 occurs due to the geometry-dependent effective thermal conductivity of a structured Si heatsink, since it disappears at *h* → 0. In this regime, the temperature increment is a linear function of the pump power, and its slope reflects directly the thermal conductance. Though eq 3 shows a linear dependence of Δ*T*_*R*_ on *h*, a FDTD/FEM simulation allows to unravel its nontrivial behavior (see Figure S2). In particular, we observe local plateaus due to the excitation of guided modes with wavelengths *k*λ/2*n*_Si_ (where *k* is a mode order and *n*_Si_ is a Si refractive index) inside the Si resonator with the open bottom end.^24,25^ These modes leak into the 3D Si thermostat and deplete the pump power. Importantly, the guided modes contribute to the optical heating insignificantly (blue circles in Figure S2), in contrast to plasmon-enhanced absorption of the TiN pad.

Herein, a side-chain 4-amino-4′-nitroazobenzene chromophore covalently attached to the poly[(methyl methacrylate) (PMMA) backbone, recognized as poly[(methyl methacrylate)-*co*-(Disperse Red 1 methacrylate)], is utilized for probing its melting point. The choice of this material is caused by strong Raman peaks originating from an azochromophore, as seen from Figure 2. The background-subtracted Raman spectrum was captured with a CW 532 nm laser light with the intensity of 120 kW/cm^2^ for 5 s, at which no heating is observed due to the absence of plasmon resonance of the TiN pads. Below we will use a CW 632.8 nm laser light which enables the 3D TiN:Si voxel to be heated. In our previous work,^13^ a poly[(methyl methacrylate) (PMMA) microstructure was chosen to develop a method for probing the glass transition temperature based on the temperature-dependent intensity of symmetric vibrational modes at 810 cm^–1^ (C–O–CH_3_) and 1460 cm^–1^ (O–CH_3_),^26^ which are marked with an asterisk in Figure 2. The azochromophore covalently attached to the PMMA backbone provides a broader set of spectral probes. Among other things, we highlight the strongest Raman peaks at 1110 and 1340 cm^–1^ assigned to the phenyl–NO_2_ stretch and the NO_2_ symmetric stretch, respectively.^27,28^ Alternative Raman bands at 1147 cm^–1^ (phenyl–NN stretch), 1195 cm^–1^ (CH in-plane bend), 1400 cm^–1^ (NN stretch), 1440 cm^–1^ (NO stretch), and 1590 cm^–1^ (CC stretch) are also suitable for quantifying the melting temperature.^27,28^ Without loss of generality, we select the Raman band at 1340 cm^–1^ (s-NO_2_ stretch) because it is the most intensive, marked with a yellow bar in Figure 2 for further analysis.

**Figure 2:**
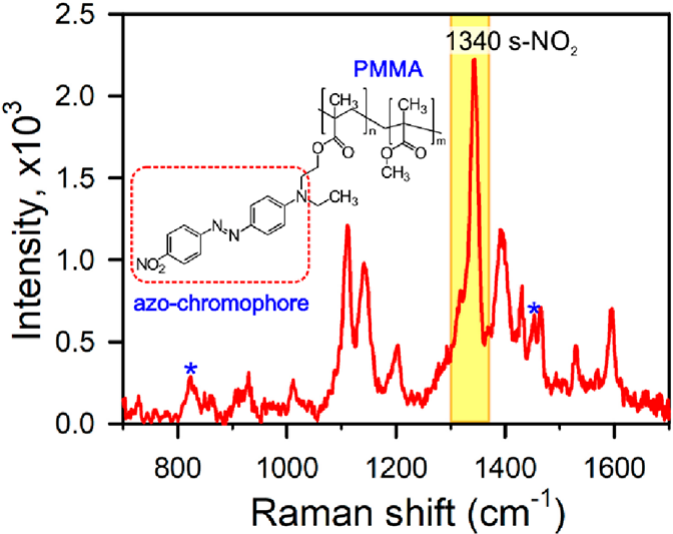
Raman spectrum of poly[(methyl methacrylate)-*co*-(Disperse Red 1 methacrylate)] under 532 nm laser illumination with the intensity of 120 kW/cm^2^ for 5 s; the inset shows a chemical structure of this molecule. The asterisks feature the Raman peaks attributed to the PMMA backbone.

Figure 3a illustrates a 52° tilted SEM image of a fabricated 2D array of TiN:Si voxels with two Si pillars heights: 100 nm (blue) and 250 nm (red) (see details in the Methods section). The TiN pad height is equal to 50 nm. Upon dropping 0.2 μL of DR1-PMMA solution on the array, a thin film forms after drying in air, as shown in Figure 3b. An analysis of the film topography using atomic force microscopy (AFM) (see Figure 3c,d) shows that its thickness is nonuniform, and it varies within the range 50–100 nm.

**Figure 3:**
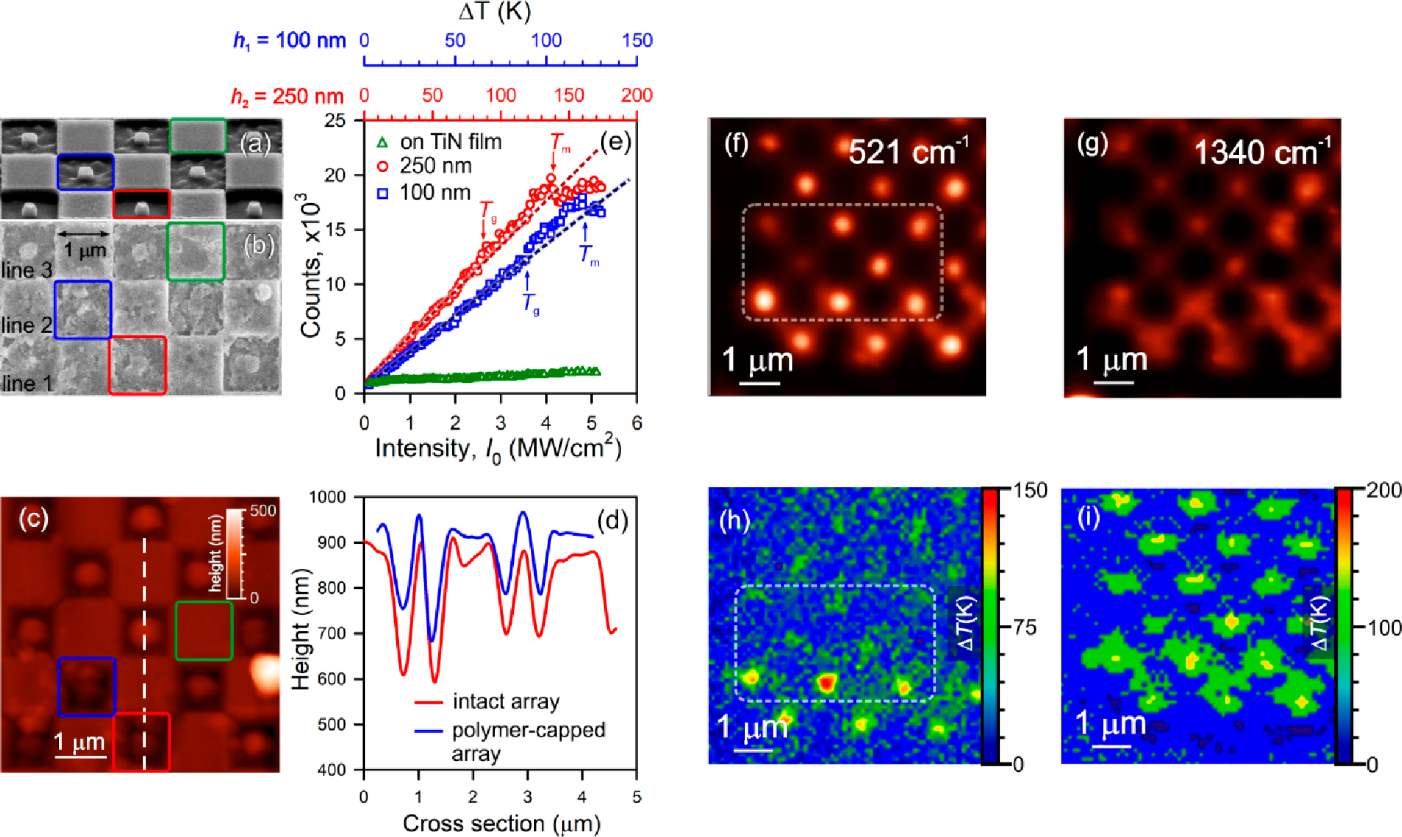
SEM image of the metasurface without (a) (under 52° tilt) and with (c) (normal view) a DR1-PMMA thin film. (c) AFM topography of the polymer-capped metasurface; (d) a cross section along a dashed straight line before (red) and after (blue) polymer deposition. (e) Plot of Raman intensity of the 1340 cm^–1^ band of DR1-PMMA deposited on TiN:Si voxels with different Si pillar heights, 100 nm (blue) and 250 nm (red), and on a TiN film (green) vs the pumping intensity. (f, g) Raman maps of a 2D array of TiN:Si voxels without and with DR1-PMMA at 521 cm^–1^ (Si) and 1340 cm^–1^ (DR1-PMMA), respectively. (h, i) Temperature maps of the metasurface exposed to 633 nm focused light with the intensity of 5 MW/cm^2^ without and with the polymer film.

The next step is to build a plot of the Raman intensity of the vibrational mode at 1340 cm^–1^ vs the pumping intensity within the range of 0–5 MW/cm^2^ at three regions, outlined in red, blue, and green (see Figure 3a–c). In the latter case, the Raman shift remains unchanged, and therefore no optical heating is observed. The Raman intensity of a polymeric structure on the TiN film (green triangles) features a linear trend with the pumping intensity, as shown in Figure 3e. Depositing a polymeric structure on a TiN:Si voxel boosts a Raman response significantly due to the enhanced absorptive cross section σ_abs_, which is directly quantified by the slope of the curves. Based on the FDTD/FEM simulation, the temperature increment of the polymer structures was calculated from a direct measurement of the Si pillar temperature by using the Raman-shift probe (see the details in the Supporting Information). The top panels in Figure 3e correspond to the temperature increments obtained with the arrays of 3D TiN:Si voxels with the different Si pillar heights: 100 nm (blue) and 250 nm (red). Figure 3 displays a Raman map at 521 cm^–1^ (Si) (f) and a 2D temperature profile of the intact array (h). A region highlighted with a dashed loop corresponds to that shown in Figures 3a,b. These results show that the longer TiN:Si voxels are heated to higher temperatures at a specific pumping intensity. A Raman map at 1340 cm^–1^ of a polymer-capped array is given in Figure 3g. It was found that the polymer is uniformly smeared near the 250 nm Si pillars, and we can declare that the polymer structures have melted. Surprisingly, a Si-based temperature map of the polymer-capped array shows uniform optical heating of all voxels regardless of the Si pillar height. In contrast, it was revealed that the optical heating is highly delocalized nearby the longer Si pillars. This removes the contradiction related to the total energy absorbed by the short and long TiN:Si voxels (see Figure 3i).

Figure 3e makes it possible to discover both the glass transition temperature *T*_g_ and the melting point *T*_m_. However, the underlying physical mechanisms to interpret the observed hallmark kinks are different. With the onset of vitrification, the vibrational modes are unfrozen due to an increase in the free volume, and therefore the population of Stockes states grows. As a result, a Raman intensity experiences an up-kink, while the opposite outcome has been previously demonstrated for a freestanding polystyrene thin film under a resistive heating.^29,30^ According to our strategy, the optical pumping is simultaneously used to heat and probe a specimen of interest. With further optical heating, this tendency disappears and the slope returns to its original value. The temperature increment at a given pump power is controlled the height of Si pillars, so both the curves provide similar results: *T*_g_ = *T*_0_ + Δ*T* = 112 ± 5 °C, where *T*_0_ is the room temperature (red circles) and *T*_g_ = 115 ± 5 °C (blue squares). The quantification of the glass transition temperature was performed by using a cumulated Pearson’s correlation method developed in ref (13). In contrast to resistive heating, the rate of optical heating is almost instantaneous, and it is less than 1 μs. It is important to note the enhanced Raman intensity at the *T*_g_ event remains open to interpretation. We propose two hypotheses: one of them is related to a reinforced segmental mobility of the backbone that is capable of enlarging the population of Stokes states. In other words, an additional intrinsic heatsink inside a sample appears, which provides the best performance of Raman scattering. The second mechanism is linked to a temperature-dependent thermal conductivity of the polymer itself that changes the uptrend for the opposite with the advent of the glass transition. In fact, both scenarios are aimed at lowering the temperature of the entire heating system—a 3D TiN:Si voxel coated with a polymer at the *T*_g_.

With further optical heating, we observe another peculiarity in the behavior of Raman intensity of the vibrational mode; namely, that it decreases sharply, as seen in Figure 3e. We attribute this characteristic fracture to the melting of the polymer. This effect can be explained through polymer spreading over the microstructure. A decrease of Raman intensity is a result of the depleted polymer on a 3D TiN:Si voxel. Figure 3b clearly shows the smearing of the polymer under CW illumination in line 2 compared to the intact polymer in line 1. Figure 3e permits us directly to monitor the melting temperature for short (blue squares) and long (red circles) TiN:Si voxels, which are equal to *T*_m_ = 145 ± 5 °C and *T*_m_ = 160 ± 5 °C, respectively. A deviation in the former case is caused by a limited temperature scale at which the polymer is not heated to the melting point well, as follows from Figure 1b. The accuracy of this method is critically dependent on the lateral size of a 3D TiN:Si voxel: the smaller the size, the higher the sensitivity. Thus, all-optical method can be used to reliably recognize the melting point of 3D confined polymers.

This work reported the first demonstration of optical heating on the nanoscale by using not only the pump power but also heat localization within a structured heatsink. A concept rationale has been performed by using a 2D array of 3D TiN:Si voxels consisting of stacked TiN pads and Si pillars, which play a role of nanoheaters and spatially limited heatsinks, respectively. The TiN nanoheaters are fueled by light at a plasmon resonance, whereas heat transfer is driven through height-controlled Si pillars. The latter allows one to provide excess heating due to heat localization. This facilitates the increase of a working temperature range, whereas the pumping intensity enables direct temperatures at settled on-demand temperature within the limits of the range. On the basis of this, we have developed a method to locally probe the first- and second-order phase transitions at the nanoscale. The power of this method has been verified through finding both the glass transition temperature, *T*_m_ = 112 ± 5 °C, and the melting temperature, *T*_m_ = 160 ± 5 °C, of 3D confined azopolymers, which are proved to be in good agreement with data from the literature. Upon reaching the melting point, *T*_m_, the melted polymer spreads over the voxel and a Raman signal decreases abruptly. A high accuracy of the method, 5 °C, has been reasoned by a Raman-shift-based probe showing a spectral resolution of 0.1 cm^–1^ due to an Echelle grating. We are confident that this concept will be beneficial to the development of diverse thermo-optical devices such as large-area broadband absorbers for solar thermophotovoltaics,^25^ ultracompact heaters for fast optical thermocycling for polymerase chain reactions,^31^ nanofurnaces for catalytic reactions,^32,33^ biointerfaces for thermally patterned neuromodulation,^34^ and others.

## Methods and Materials

### Sample Preparation

Poly[(methyl methacrylate)-*co*-(Disperse Red 1 methacrylate)] (Sigma-Aldrich), abbreviated as DR1-PMMA, was used as a test specimen for probing both the glass transition temperature *T*_g_ = 108 °C (by DSC) and the melting temperature *T*_m_. The original 1 wt % DR1-PMMA granules were dissolved in 5 mL of chloroform. The solvent was of analytical grade and purchased from Merck. A 0.2 μL DR1-PMMA droplet was put on a 2D array of TiN:Si voxels. A polymer thin film forms after drying in air.

### Atomic Force Microscopy Measurements

The thickness of the DR1-PMMA film was determined by starching its surface with a multimode scanning probe microscope Prima (NT-MDT), as reported in ref (35). The depth of lithographically fabricated grooves on the DR1:PMMA film was controlled by the set point driven applied force of the c-AFM cantilever. The optimal set point was found upon reaching a force-independent depth. The probes of the “Etalon” series with resonant frequencies around 70 and 110 kHz were used in AFM measurements. A topography of the DR1-PMMA thin film before and after its light-induced melting was monitored by using AFM.

### Synthesis, Nanofabrication, and Characterization of a Titanium Nitride (TiN) Thin Film

TiN thin films on c-Si (100) substrates were synthesized by using direct current reactive magnetron sputtering (a power of 200 W) in the Ar/N_2_ environment with a volume proportion of 30:70 at elevated temperature of 350 °C and a base pressure of 3 × 10^–9^ mbar. Prior to the film growth, the c-Si substrate was sonicated in acetone for 15 min. The thickness of the TiN films, equal to 50 ± 5 nm, was measured with a contact profilometer Alpha Step 200. Square-shaped TiN pads caped the c-Si nanopillars were engraved through focused ion beam milling at a lower current of 1 pA by using Quanta 3D FEG (FEI). 2D visualization of thermal metasurfaces was performed with scanning electron microscopy (Quanta 3D FEG (FEI)).

### Raman Spectroscopy and Microscopy

Far-field Raman spectra and maps were registered with a multipurpose analytical instrument NTEGRA SPECTRA (NT-MDT) in the upright configuration. The confocal spectrometer was wavelength calibrated with a crystalline silicon (100) wafer by registering the first-order Raman band at 521 cm^–1^. A sensitivity of the spectrometer was as high as ca. 14000 photon counts per 0.1 s provided that we used a 100× objective (N.A. = 0.9), a pinhole of 100 μm, and linearly polarized light with a wavelength of 632.8 nm and a power at the sample of 15 mW. No signal amplification regimes of a Newton EMCCD camera (ANDOR) were used. The 128 × 128 pixel Raman maps were raster scanned with a step of 40 nm and an exposure time per pixel of 0.1 s and were finally collected with the EMCCD camera cooled to −95 °C. Raman spectra were registered with a spectral resolution of 0.1 and 1.3 cm^–1^ by using the Echelle and 600 grooves/mm gratings, respectively.

### FDTD/FEM Calculation

3D simulation of the absorption in a square-shaped TiN pad-capped a c-Si (111) nanopillar under CW illumination was performed by using an Ansys/Lumerical FDTD solver. The height of the TiN pad was 50 nm, whereas the height of the c-Si nanopillar varied within the range 0–250 nm (see the geometry in Figure 1a). To suppress anomalous electric fields near the edges and the corners of the TiN pad, we used structures with rounded edges and corners (10 nm rounding). A mesh overlayer of 1 nm was utilized around the TiN pad and a rougher 5 nm mesh for the rest of the structure. Perfectly matching layers were used as boundary conditions for three directions. The optical and thermal properties of the c-Si and air were imported from the Ansys/Lumerical material database. The TiN pad was exposed to a 632.8 nm focused laser light (NA = 0.9) with an intensity of 15 MW/cm^2^. The temperature profile was calculated through an Ansys/Lumerical FEM solver in the steady-state regime. The thermal conductivity of all constituents is assumed to be temperature-independent. The boundary condition of *T* = 300 K was set at the *z*_min_ = −3500 nm of the 20 × 20 × 5 μm^3^ simulation region.

## Supporting Information Available

Theoretical details on a derivation of eq 3; a calibration plot for Raman shift-based Si probe; FDTD/FEM simulation of temperature of a TiN:Si voxel vs Si pillar height (PDF)
