# Supplementary material for: Nanoscale Melting of 3D Confined Azopolymers through Tunable Thermoplasmonics
Source: J Phys Chem Lett. 2022 Jun 9;13(23):5351–7. doi: 10.1021/acs.jpclett.2c01103 (PMC9208006; doi:10.1021/acs.jpclett.2c01103)
Supplement: Supplementary file 1 — jz2c01103_si_001.pdf [file jz2c01103_si_001.pdf]

Supporting Information: Nanoscale Melting of 3D Confined Azo-Polymers Through Tunable Thermoplasmonics

Sergey S. Kharintsev[1] and Sergei G. Kazarian[2]**

[1]Department of Optics and Nanophotonics, Institute of Physics, Kazan Federal University, Kremlevskaya, 16, Kazan, 420008, Russia

[2]Department of Chemical Engineering, Imperial College London, South Kensington Campus, SW7 2AZ, United Kingdom

1. An analytical derivation of equation (2).

In the steady-state regime, the distribution of temperature $T(\boldsymbol{r})$ in space is governed by the thermal diffusion equation

$$\nabla[k(\boldsymbol{r},T)\nabla T(\boldsymbol{r})] = -q(\boldsymbol{r}), \tag{S1}$$

which reflects a balance between a total absorbed energy

$$Q = \int_V q(\boldsymbol{r})d\boldsymbol{r} \equiv \sigma_{\text{abs}} I_0 \sim \frac{V\,\text{Im}[\varepsilon_T] I_0}{\lambda}, \tag{S2}$$

(where $q(\boldsymbol{r})$ is a heat power density, λ is the wavelength of incident light, σ_{abs} is an absorption cross section, V is a structure volume, ε_T is the temperature-dependent permittivity of a structure, I_0 is the pumping intensity) and heat dissipation towards the surrounding, which is described by its position- and temperature-dependent thermal conductivity $k_T(\boldsymbol{r})$. In the case of

plasmon resonance, metallic nanospheres serve as perfect thermal nano-sources due to enhanced inner electric fields, which are responsible for heat generation. In the quasi-static approximation, Eq. (S2) is modified as follows:

$$Q \sim \frac{V\,\mathrm{Im}[\varepsilon]}{\lambda} g^2 I_0, \tag{S3}$$

in which we introduced the field enhancement factor $g(\lambda)$ defined as[1]

$$g(\lambda) = \left| \frac{3\varepsilon_s}{\varepsilon(\lambda) + 2\varepsilon_s} \right|, \tag{S4}$$

here ε_s is the surrounding permittivity. Local density of states (LDOS) amplification at the l-th order dipole plasmon resonance subject to the Fröhlich condition: $\mathrm{Re}[\varepsilon(\lambda_{\mathrm{res}})] = -[(l+1)/l]\varepsilon_s$ (λ_{res} is the resonant wavelength), yields

$$g^2(\lambda_{\mathrm{res}}) = 9\left(\frac{l}{l+1}\right)^2 \left\{ -\frac{\mathrm{Re}[\varepsilon(\lambda_{\mathrm{res}})]}{\mathrm{Im}[\varepsilon(\lambda_{\mathrm{res}})]} \right\}^2, \tag{S5}$$

where in braces we find the usual figure of merit widely used in plasmonics.[2] From Eq. (S5) follows that the highest-order plasmon modes excited inside a metallic nanostructure with ε_m satisfying an inequality: $\mathrm{Re}[\varepsilon] > \mathrm{Im}[\varepsilon]$, contribute into the optical heating strongly (several orders of magnitude).

Under the lowest-order dipole plasmon resonance subject to the Fröhlich condition: $\mathrm{Re}[\varepsilon(\lambda_{\mathrm{res}})] = -2\varepsilon_s$ (λ_{res} is the resonant wavelength), a temperature rise of the metallic nanostructure exposed to cw laser illumination reads[3,4]

$$\Delta T_R = \frac{3}{4}\frac{R^2}{\beta\lambda_{\mathrm{res}}}\frac{\mathrm{Re}[\varepsilon_T(\lambda_{\mathrm{res}})]^2}{\mathrm{Im}[\varepsilon_T(\lambda_{\mathrm{res}})]}\frac{I_0}{\kappa_T}, \tag{S6}$$

here β is a dimensionless thermal capacitance coefficient, depending on the nanostructure geometry, R is the radius of a sphere with the same volume as the nanostructure.[17] Under

resonance, the heating of a nanostructure is controlled by the pump power I_0 and the thermal conductivity κ_T only. As follows from Eq. (S6), the optical heating of a nanostructure surrounded by a high thermal conductivity thermostat is negligible regardless of a pumping intensity. At a fixed pump power, the tunable optical heating can be performed through heat localization within a spatially-limited designed heatsink. For this purpose we place a nano-heater on a rod-shaped heatsink of height h. Upon expanding $\kappa_T(\boldsymbol{r})$ as a function of position $\boldsymbol{r}$ into a Taylor series and limiting to the first two terms, Eq. (S6) can be modified as follows

$$\Delta T_R(h) \approx \frac{3}{4}\frac{R^2}{\beta\lambda_{\text{res}}}\frac{\text{Re}[\varepsilon(\lambda_{\text{res}})]^2}{\text{Im}[\varepsilon(\lambda_{\text{res}})]}\left[1-\frac{\nabla_z\kappa_T(\boldsymbol{r})}{\kappa_T(0)}h\right]\frac{I_0}{\kappa_T(0)}, \tag{S7}$$

where $\kappa_T(0)$ is the temperature-dependent thermal conductivity at $h=0$, $\nabla_z \equiv \partial/\partial z$ is the z-projection of the Laplace operator. The gradient $\nabla_z\kappa_T$ has a negative sign because the $\partial k_T/\partial T < 0$ for the most materials at room temperature. Upon applying the Fourier law, we have

$$\nabla_z\kappa_T = \frac{\partial k_T}{\partial T}\nabla_z T \equiv \frac{\partial \ln(k_T)}{\partial T}\sigma_{\text{abs}}I_0. \tag{S8}$$

Finally, we derive

$$\Delta T_R(h) \approx \frac{3}{4}\frac{R^2}{\beta\lambda_{\text{res}}}\frac{\text{Re}[\varepsilon(\lambda_{\text{res}})]^2}{\text{Im}[\varepsilon(\lambda_{\text{res}})]}\left[\frac{I_0}{\kappa_T(0)}-\frac{\sigma_{\text{abs}}h}{\kappa_T^3(0)}\frac{\partial k_T}{\partial T}I_0^2\right]. \tag{S9}$$

2. A calibration plot based on the temperature-dependent band shift

Raman scattering represents a powerful instrument for remote measuring a temperature. Since the lattice spacing is sensitive to temperature, it leads to the Raman peak shift and, therefore, the latter can be used as a temperature probe. For most materials, the specific Stokes

peaks are red-shifted with increasing temperature. The Stokes peak shift $\Delta(T)$ as a function of T is determined as follows[1,2]

$$\Delta(T) = \Omega(T) - \Omega(T_0) = A\left(1 + \frac{2}{e^{\frac{\hbar\omega_0}{2kT}} - 1}\right) + B\left(1 + \frac{3}{e^{\frac{\hbar\omega_0}{3kT}} - 1} + \frac{3}{(e^{\frac{\hbar\omega_0}{3kT}} - 1)^2}\right) \qquad \text{(S10)}$$

where A and B are constants specific to materials, ω_0 is the incident photon frequency, Ω is a phonon frequency, $\hbar$ is the Plank's constant, k_B is the Boltzmann's coefficient, T is the absolute temperature of a sample in unit of K, T_0 is equal to 0 K. For silicon, we found the

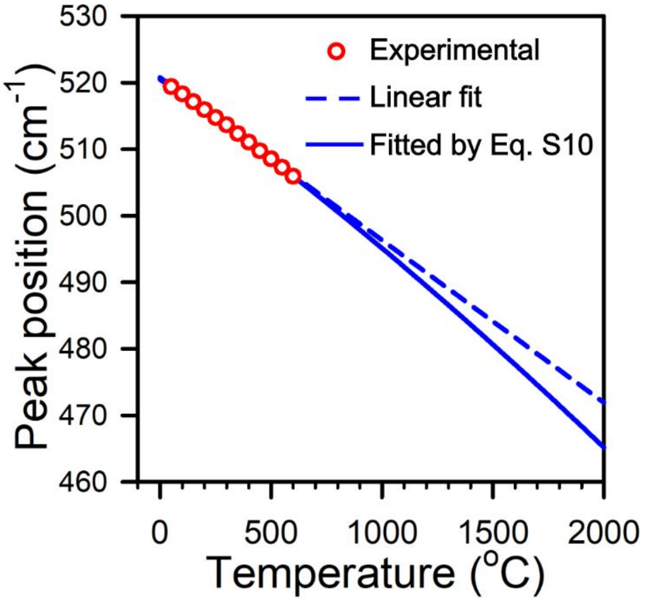

Figure S1. A plot of the peak position vs the temperature for pure silicon.

following values $A = -4.391\ \text{cm}^{-1}$ and $B = -0.042\ \text{cm}^{-1}$ using temperature-dependent Raman measurements in the range from 25°C to 600°C (see Fig. S1). As seen from the figure, the experimental data can be reliably fitted by a linear function within the range from 25°C to 200°C (dashed blue curve). For larger temperatures, these should be extrapolated by using Eq. S10 (solid blue curve).

3. FDTD/FEM simulation of temperature of a TiN:Si voxel vs Si pillar height

In FDTD/FEM simulation, the 3D TiN:Si voxel with the lateral size of 200 nm was illuminated by a focused 633 nm laser light with the intensity of 5 MW/cm^2. We have calculated the temperature rise as a function of the Si pillar height when light is absorbed by the TiN pad (green), the Si pillar (blue) and the entire TiN:Si structure (red) separately, as outlined with the color in the inset of Fig. S2. The calculated curves for both the TiN:Si and the TiN do not follow a linear law and exhibit local plateaus due to the excitation of guided modes with wavelengths $k\lambda/2n_{\mathrm{Si}}$ (where k is a mode order, n_{Si} is a Si refractive index) inside the Si resonator with the open bottom end. Eventually, these modes leak into the 3D Si thermostat and, deplete the pump power. This means that the Si pillar serves as a low Q-factor optical antenna. However, the guided modes contribute to the optical heating insignificantly (blue circles in Fig. S2) and that is mainly caused by the volume V and the imaginary part of permittivity ε (see Eq. S2). While the TiN pad itself contributes into the optical heating at a plasmon resonance larger. Summarizing, the heat 3D TiN:Si voxel power is directly dependent on the height of the Si pillar. In a rough approximation, one can claim that the temperature increment is driven by the height linearly.

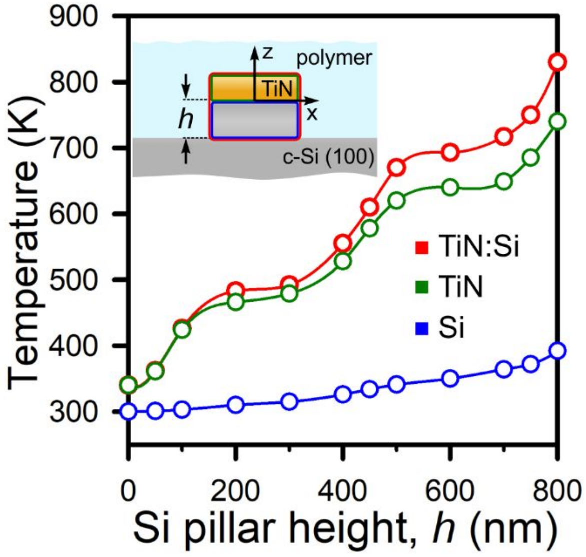

Figure S2. FDTD/FEM simulation of temperature vs the Si pillar height (blue, green and red open circles correspond to optical heating when the pumping intensity of 5 MW/cm^2 absorbed by Si, TiN and TiN:Si constituents, respectively). The inset displays a schematic of a 3D TiN:Si voxel in which each of the constituents that absorbs light energy is circled with the corresponding color.

References

(1) Lalisse, A.; Tessier, G.; Plain, J.; Baffou, G. Quantifying the efficiency of plasmonic materials for near-field enhancement and photothermal conversion. *J. Phys. Chem. C* **2015**, *119*, *45*, 25518–25528.

(2) Reddy, H.; Guler, U.; Kudyshev, Z.; KIldishev, A.V.; Shalaev, V.M.; Boltasseva A.E.; Temperature-dependent optical properties of plasmonic titanium nitride thin films. *ACS Photonics* **2017**, *4* (6), 1413–1420.

(3) Baffou, G.; Quidant, R.; García de Abajo, F. J. Nanoscale Control of Optical Heating in Complex Plasmonic Systems. *ACS Nano* **2010**, *4*, 709–716.

(4) Govorov, A. O.; Richardson, H. H. Generating Heat with Metal Nanoparticles. *Nano Today* **2007**, *2*, 30–38.

(5) Hart, T. R.; Aggarval, R. L.; Lax, B. Temperature Dependence of Raman Scattering in Silicon. *Phys. Rev. B* **1970**, *1* (2), 638–642.

(6) Balkanski, M.; Wallis, R. F.; Haro, E. Anharmonic Effects in Light-Scattering due to Optical Phonons in Silicon. *Phys. Rev. B.* **1983**, *28* (4), 1928–1934.
